# Depression and anxiety in women with idiopathic intracranial hypertension compared to migraine: A matched controlled cohort study

**DOI:** 10.1111/head.14465

**Published:** 2023-02-07

**Authors:** Susan P. Mollan, Anuradhaa Subramanian, Mary Perrins, Krishnarajah Nirantharakumar, Nicola J. Adderley, Alexandra J. Sinclair

**Affiliations:** ^1^ Translational Brain Science, Institute of Metabolism and Systems Research University of Birmingham Birmingham UK; ^2^ Health Data Research UK Birmingham UK; ^3^ Birmingham Neuro‐Ophthalmology Queen Elizabeth Hospital Birmingham UK; ^4^ Institute of Applied Health Research University of Birmingham Birmingham UK; ^5^ Department of Neurology University Hospitals Birmingham, Queen Elizabeth Hospital Birmingham UK; ^6^ Centre for Endocrinology, Diabetes, and Metabolism Birmingham Health Partners Birmingham UK

**Keywords:** anxiety, depression, epidemiology, idiopathic intracranial hypertension, migraine, primary care

## Abstract

**Objective:**

To evaluate mental health burden in women with idiopathic intracranial hypertension (IIH) compared to matched women with migraine and population controls.

**Background:**

Depression and anxiety are recognized comorbid conditions in those with IIH and lead to worse predicted medical outcomes. The mental health burden in IIH has not been previously evaluated in a large, matched cohort study.

**Methods:**

We performed a population‐based matched, retrospective cohort study to explore mental health outcomes (depression and anxiety). We used data from IQVIA Medical Research Data, an anonymized, nationally representative primary care electronic medical records database in the United Kingdom, from January 1, 1995, to September 25, 2019. Women aged ≥16 years were eligible for inclusion. Women with IIH (exposure) were matched by age and body mass index with up to 10 control women without IIH but with migraine (migraine controls), and without IIH or migraine (population controls).

**Results:**

A total of 3411 women with IIH, 30,879 migraine controls and 33,495 population controls were included. Of these, 237, 2372 and 1695 women with IIH, migraine controls and population controls, respectively, developed depression during follow‐up, and 179, 1826 and 1197, respectively, developed anxiety. There was a greater hazard of depression and anxiety in IIH compared to population controls (adjusted hazard ratio [aHR] 1.38, 95% confidence interval [CI] 1.20–1.58; and aHR 1.40, 95% CI 1.19–1.64, respectively), while hazards were similar to migraine controls (aHR 0.98, 95% CI 0.86–1.13; and aHR 0.98, 95% CI 0.83–1.14, respectively).

**Conclusion:**

Depression and anxiety burden in women with IIH is higher than in the general population, and comparable to that in matched women with migraine. This may indicate that presence of headache is a potential driver for comorbid depression and anxiety in IIH.

**Abbreviations**
aHR: adjusted hazard ratio
BMI: body mass index
CI: confidence interval
DExtER: Data Extraction for Epidemiological Research
HR: hazard ratio
ICP: intracranial pressure
IHR: Intracranial Hypertension Registry
IIH: idiopathic intracranial hypertension
IMRD: IQVIA Medical Research Data
N/A: not applicable
NPI‐Q: Neuropsychiatric Inventory Questionnaire
SD: standard deviation
THIN: The Health Improvement Network

## INTRODUCTION

Idiopathic intracranial hypertension (IIH) has become an increasingly recognized condition.^1^, ^2^, ^3^ It is characterized by raised intracranial pressure (ICP) that causes headaches and papilloedema with no direct identifiable cause.^4^, ^5^ It has clear diagnostic criteria that include normal neuroimaging (with typical signs of raised ICP being noted) and venography and a lumbar puncture opening pressure >25 cm cerebrospinal fluid in a properly performed procedure.^6^ IIH has an established association with obesity and recent weight gain is a key risk factor for developing the disease.^7^, ^8^, ^9^ There is an increasing incidence and prevalence of IIH.^1^, ^2^, ^10^

While depression and anxiety have long been known to be associated with IIH,^11^, ^12^ there have been more modern evaluations both in adult and pediatric populations.^13^, ^14^, ^15^, ^16^, ^17^ In one study which surveyed a large patient group, up to 56% had depression as measured by the Major Depression Inventory, with one‐third in the survey actively taking antidepressant medicines.^15^ Using the Neuropsychiatric Inventory Questionnaire (NPI‐Q) in a small cohort, 86% reported psychiatric symptoms, with 83% having depression‐anxiety syndromes.^16^ More concerning was the Intracranial Hypertension Registry (IHR) cohort that found, compared to the general population, the risk of suicide was over six‐times greater and the risk of death from accidental overdose was over three‐times greater in IIH. The risk of suicide by overdose was over 15‐times greater among the IHR cohort than in the general population.^17^ However, mental health is also known to be affected in migraine,^18^, ^19^ and as the majority of those with IIH have migraine‐like headaches, we sought to understand whether the burden of mental health in IIH was different to people with migraine.

We hypothesized that there may be an increased risk of depression and anxiety in women with IIH compared to women without IIH. The aim of this study was therefore to evaluate the incidence of depression and anxiety in women with IIH, and to determine whether this was different in women with IIH compared to control women with and without migraine.

## METHODS

### Study design

We performed an age‐ and body mass index (BMI)‐matched retrospective open cohort study using data from January 1, 1995, to September 25, 2019. The start date of 1995 was selected to ensure good data quality; end date was the date of the latest available data at the time of data extraction.

### Data source

Study data were extracted from IQVIA Medical Research Data (IMRD‐UK), which incorporates data from The Health Improvement Network (THIN), a national database of electronic primary care records that is generalizable to the UK population. IMRD‐UK contains anonymized, coded information for >15 million patients from >800 primary care general practices, including patient demographics, symptoms, diagnoses, drug prescriptions, consultations, and laboratory test results.^20^ In the UK, an IIH diagnosis is made following diagnostic investigations performed in a hospital setting, and the diagnosis is then communicated to general practice or primary care physicians and recorded in the patient's primary care record; this process increases the likelihood that the diagnosis in the primary care electronic record is correct. Diagnoses of the mental health outcomes (depression and anxiety) may be made in primary or secondary care; where the diagnosis is made in secondary care, this would be communicated to the general practice and recorded in the patient's primary care record. Studies utilizing IMRD/THIN data for IIH analysis have previously been published.^1^ The study dataset was extracted from the IMRD database using the Data Extraction for Epidemiological Research (DExtER) tool.^21^

### Population

To maximize data quality, general practices were eligible for inclusion in the study from the latest of the following two dates: 12 months after the date from which they reported acceptable mortality recording rates^22^ and 12 months after the practice began using electronic medical records. Adult women aged ≥16 years and registered with any eligible general practice for a minimum of 365 days were included in the study.

#### Inclusion criteria for women with IIH (exposed group)

A diagnosis of IIH was defined using a record of a clinical (Read)^23^ code for IIH in the patient's primary care records (Table S1A). All women with IIH who also had a clinical code for hydrocephalus or cerebral venous sinus thrombosis were excluded in case they had been miscoded (Table S1B,C). To assess rates of mental health outcomes (depression and anxiety), women with prevalent and incident IIH (and controls) were included; in a sensitivity analysis only women with incident IIH (and their corresponding controls) were included. IIH is a rare disease and therefore all eligible women with IIH in the database were included; the sample size was based on the available data and no a priori statistical power calculation was conducted.

#### Inclusion criteria for migraine controls (unexposed)

For each woman with IIH, up to 10 controls were selected from a pool of women with a clinically coded migraine diagnosis (Table S1D) but no IIH. Propensity scores were used to select closely matched migraine control women. Propensity scores were estimated using a logistic regression model with a caliper of width 0.2; index date, age, BMI category, Townsend deprivation quintile, ethnicity and smoking status were included as independent variables in the model.

#### Inclusion criteria for population controls (unexposed)

For each woman with IIH, up to 10 population control women without a record of IIH or migraine were included. Population control women were randomly selected from a pool of women directly matched to the IIH population on the index date by age (±1 year) and BMI (±2 kg/m^2^). Matching (without replacement) was undertaken using DExtER.^21^ Controls with a diagnosis of hydrocephalus or cerebral venous sinus thrombosis were excluded.

### Follow‐up period

For patients with incident IIH (newly diagnosed during the study period), index date was the date of IIH diagnosis. For patients with prevalent IIH (with a pre‐existing diagnosis at study entry), the index date was the latest of the following two dates: 1 year after registration with the general practice or 1 year after the date the practice became eligible to take part in the study, to ensure sufficient time for important baseline comorbidities to be recorded. Population controls were assigned the same index date as their corresponding patient with IIH to mitigate immortal time bias.^24^ For migraine controls, index date was the date of migraine diagnosis; index year was included among the propensity score matching variables.

All patients were followed‐up from their index date until the earliest of the following events: outcome (depression, anxiety), death, patient left the practice, practice ceased contributing to the database, or study end (September 25, 2019).

### Outcomes

The primary outcomes were a new diagnosis of depression or anxiety. The outcomes were defined by the presence of a relevant clinical (Read) code in the primary care record (Table S1E,F). Incident mental health outcomes were compared between women with IIH and both the migraine control group and the population control group.

### Analysis

Baseline information was reported as number (%) for categorical data and as mean (standard deviation [SD]) for continuous numerical variables. Incidence rates (IRs) for depression and anxiety per 1000 person‐years were calculated in women with a record of IIH (prevalent or incident) and in the migraine control group and population control group. Cox proportional hazards regression was used to calculate crude hazard ratios (HRs) and adjusted HRs (aHRs) and their corresponding 95% confidence intervals (CIs) for rates in women with IIH compared to both control groups for both outcomes. For each outcome, patients with a record of the outcome at baseline were excluded. Regression models were adjusted for age category, BMI category, Townsend deprivation quintile, smoking status (categorized as non‐smoker, current smoker, and ex‐smoker), eating disorder, severe mental illness (schizophrenia, bipolar disorder, psychosis, paranoid ideation, manic disorders, and delusional disorders), back pain, osteoarthritis, rheumatoid arthritis, fibromyalgia, epilepsy, obstructive sleep apnea, and polycystic ovary syndrome. The proportional hazards assumption was checked using the Schoenfeld residuals test. Cumulative hazards were plotted for depression and anxiety in women with IIH, migraine controls and population controls.

A sensitivity analysis was carried out limiting the analysis to patients with incident IIH (newly diagnosed during the study period) and their corresponding controls to explore any impact of survival bias.

All analyses were performed in Stata IC version 16. Two‐sided *p*‐values were obtained, with a *p* < 0.05 considered statistically significant.

### Missing data

Missing data for BMI, Townsend deprivation quintile, ethnicity and smoking status were included in a separate missing category in propensity score matching and the Cox regression models. Exposed patients with missing BMI were matched to population controls with missing BMI. Absence of a clinical code for a disease was interpreted as indicating absence of the corresponding disease.

## RESULTS

### Baseline characteristics

A total of 3411 women with IIH, 30,879 migraine controls and 33,495 controls were included in the analysis (Figure S1). Baseline characteristics are presented in Table 1 (see also Table S2). The mean age in all three groups was 34 years. The mean (SD) BMI was 34.9 (8.1), 28.8 (6.3) and 34.4 (7.8) kg/m^2^ in the IIH, migraine control and population control groups, respectively. The proportion of participants with several comorbidities, including back pain, polycystic ovary syndrome, osteoarthritis, epilepsy, fibromyalgia, sleep apnea and severe mental illness, was higher in women with IIH compared to both control groups (Table 1).

**TABLE 1:** Baseline characteristics of exposed participants with idiopathic intracranial hypertension (IIH) and matched controls with migraine (but without IIH), and matched population controls (with neither IIH nor migraine).

Characteristic	Primary analysis			Sensitivity analysis (incident IIH and corresponding controls)		
	Exposed (*n* = 3411)	Migraine controls (*n* = 30,879)	Population controls (*n* = 33,495)	Exposed (*n* = 1555)	Migraine controls (*n* = 13,966)	Population controls (*n* = 15,265)
IIH duration (prevalent patients), years, mean (SD)	8.5 (8.9)	N/A	N/A	N/A	N/A	N/A
Age at IIH diagnosis, years, mean (SD)	29.4 (11.5)	N/A	N/A	32.1 (11.4)	N/A	N/A
Age, years, mean (SD)	34.0 (12.7)	33.7 (11.8)	34.1 (12.7)	32.1 (11.4)	31.9 (10.7)	32.2 (11.4)
Age categories, years, *n* (%)						
16–30	1568 (46.0)	14,143 (45.8)	15,227 (45.5)	814 (52.3)	7328 (52.5)	7903 (51.8)
30–40	940 (27.6)	8668 (28.1)	9308 (27.8)	419 (26.9)	3771 (27.0)	4167 (27.3)
40–50	499 (14.6)	4760 (15.4)	4955 (14.8)	199 (12.8)	1891 (13.5)	1988 (13.0)
50–60	262 (7.7)	2403 (7.8)	2619 (7.8)	84 (5.4)	734 (5.3)	822 (5.4)
60–70	92 (2.7)	716 (2.3)	876 (2.6)	24 (1.5)	190 (1.4)	234 (1.5)
>70	50 (1.5)	189 (0.6)	510 (1.5)	15 (1.0)	52 (0.4)	151 (1.0)
BMI, kg/m^2^, mean (SD)	34.9 (8.1)	28.8 (6.3)	34.4 (7.8)	35.7 (8.1)	29.4 (6.4)	35.1 (7.7)
BMI categories, kg/m^2^, *n* (%)						
Underweight (<18.5)	15 (0.4)	275 (0.9)	130 (0.4)	7 (0.45)	86 (0.6)	48 (0.3)
Normal weight (18.5–25)	311 (9.1)	5737 (18.6)	3364 (10.0)	111 (7.1)	2096 (15.0)	1299 (8.5)
Overweight (25–30)	503 (14.7)	8856 (28.7)	5181 (15.5)	232 (14.9)	4047 (29.0)	2365 (15.5)
Obese (>30)	2011 (59.0)	8296 (26.9)	19,194 (57.3)	1023 (65.8)	4121 (29.5)	9749 (63.9)
Missing	571 (16.7)	7715 (25.0)	5626 (16.8)	182 (11.7)	3616 (25.9)	1804 (11.8)
Smoking status, *n* (%)						
Non‐smoker	1657 (48.6)	16,308 (52.8)	18,771 (56.0)	789 (50.7)	7353 (52.6)	8769 (57.4)
Ex‐smoker	584 (17.1)	4791 (15.5)	5064 (15.1)	267 (17.2)	2197 (15.7)	2314 (15.2)
Smoker	976 (28.6)	7747 (25.1)	7298 (21.8)	457 (29.4)	3650 (26.1)	3467 (22.7)
Missing	194 (5.7)	2033 (6.6)	2362 (7.1)	42 (2.7)	766 (5.5)	715 (4.7)
Townsend deprivation quintile, *n* (%)						
1 (least deprived)	439 (12.9)	4805 (15.6)	4895 (14.6)	201 (12.9)	1991 (14.3)	2066 (13.5)
2	434 (12.7)	4543 (14.7)	4972 (14.8)	188 (12.1)	2023 (14.5)	2162 (14.2)
3	601 (17.6)	5616 (18.2)	5882 (17.6)	277 (17.8)	2529 (18.1)	2672 (17.5)
4	619 (18.1)	5423 (17.6)	6077 (18.1)	282 (18.1)	2518 (18.0)	2807 (18.4)
5 (most deprived)	515 (15.1)	4103 (13.3)	4818 (14.4)	262 (16.8)	1939 (13.9)	2285 (15.0)
Missing	803 (23.5)	6389 (20.7)	6851 (20.5)	345 (22.2)	2966 (21.2)	3273 (21.4)
Ethnicity, *n* (%)						
White	1766 (51.8)	15,389 (49.8)	14,464 (43.2)	754 (48.5)	7181 (51.4)	7009 (45.9)
South Asian	31 (0.9)	447 (1.4)	640 (1.9)	19 (1.2)	195 (1.4)	303 (2.0)
Black Afro‐Caribbean	60 (1.8)	484 (1.6)	584 (1.7)	33 (2.1)	242 (1.7)	298 (2.0)
Mixed race	15 (0.4)	162 (0.5)	249 (0.7)	6 (0.4)	73 (0.5)	117 (0.8)
Chinese/Middle Eastern/Other	15 (0.4)	156 (0.5)	171 (0.5)	5 (0.3)	76 (0.5)	80 (0.5)
Missing	1524 (44.7)	14,241 (46.1)	17,387 (51.9)	738 (47.5)	6199 (44.4)	7458 (48.9)
Comorbidities, *n* (%)						
Back pain	780 (22.9)	6522 (21.1)	5747 (17.2)	418 (26.9)	2747 (19.7)	2491 (16.3)
Polycystic ovary syndrome	253 (7.4)	1223 (4.0)	1652 (4.9)	123 (7.9)	621 (4.4)	858 (5.6)
Osteoarthritis	138 (4.0)	914 (3.0)	1022 (3.1)	42 (2.7)	287 (2.1)	385 (2.5)
Epilepsy	90 (2.6)	603 (2.0)	583 (1.7)	32 (2.1)	294 (2.1)	253 (1.7)
Fibromyalgia	69 (2.0)	354 (1.1)	215 (0.6)	36 (2.3)	170 (1.2)	110 (0.7)
Eating disorder	63 (1.8)	514 (1.7)	350 (1.0)	26 (1.7)	241 (1.7)	172 (1.1)
Severe mental illness	55 (1.6)	238 (0.8)	340 (1.0)	27 (1.7)	115 (0.8)	143 (0.9)
Obstructive sleep apnea	34 (1.0)	95 (0.3)	181 (0.5)	24 (1.5)	39 (0.3)	99 (0.6)
Rheumatoid arthritis	21 (0.6)	154 (0.5)	166 (0.5)	10 (0.6)	48 (0.3)	76 (0.5)
Outcomes at baseline, *n* (%)^a^						
Anxiety	427 (12.5)	3855 (12.5)	3157 (9.4)	223 (14.3)	1715 (12.3)	1429 (9.4)
Depression	899 (26.4)	7097 (23.0)	6076 (18.1)	463 (29.8)	3253 (23.3)	2830 (18.5)

Abbreviations: BMI, body mass index; IIH, idiopathic intracranial hypertension; N/A, not applicable; SD, standard deviation.

^a^ Women in the included cohort who had a record of either of depression or anxiety (outcomes of interest) at baseline. These women were excluded from the corresponding analysis (e.g., women with depression at baseline were excluded from the analysis exploring incident depression outcomes).

### Depression and anxiety outcomes (IIH, migraine controls, and population controls)

A total of 2512 women with IIH, 23,782 migraine controls and 27,419 population controls were included in the analysis for new onset depression. The crude incidence of depression was 20.1, 19.5 and 13.3 per 1000 person‐years in IIH, migraine controls and population controls, respectively (Table 2). Compared to migraine controls, the aHR for depression in women with IIH was 0.98 (95% CI 0.86–1.13); compared to population controls, the aHR was 1.38 (95% CI 1.20–1.58). A sensitivity analysis restricted to only women with incident IIH and their corresponding controls made little difference to these results (aHR 1.02, 95% CI 0.83–1.26; and 1.44, 95% CI 1.16–1.78 for IIH compared to migraine controls and population controls, respectively).

**TABLE 2:** Incidence rates and hazard ratios of new onset depression and anxiety in women with idiopathic intracranial hypertension (IIH) compared to matched controls with migraine and matched population controls.

	Primary analysis			Sensitivity analysis^a^		
	Exposed	Migraine controls	Population controls	Exposed	Migraine controls	Population controls
Depression						
Number of patients, *N*	2512	23,782	27,419	1092	10,713	12,435
Outcome events, *n* (%)	237 (9.4)	2372 (10.0)	1695 (6.2)	101 (9.2)	1084 (10.1)	686 (5.5)
Person‐years	11,773	121,762	127,795	4529	50,968	49,135
Crude incidence rate/1000 person‐years	20.1	19.5	13.3	22.3	21.3	14.0
Unadjusted hazard ratio (95% CI)		1.07 (0.94–1.23), *p* = 0.303	1.51 (1.31–1.73), *p* < 0.001		1.14 (0.93–1.40), *p* = 0.220	1.59 (1.29–1.96), *p* < 0.001
Adjusted hazard ratio^b^ (95% CI)		0.98 (0.86–1.13), *p* = 0.816	1.38 (1.20–1.58), *p* < 0.001		1.02 (0.83–1.26), *p* = 0.835	1.44 (1.16–1.78), *p* < 0.001
Anxiety						
Number of patients, *N*	2984	27,024	30,338	1332	12,251	13,836
Outcome events, *n* (%)	179 (6.0)	1826 (6.8)	1197 (3.9)	78 (5.9)	839 (6.8)	544 (3.9)
Person‐years	14,405	145,246	145,701	5759	61,339	55,921
Crude incidence rate/1000 person‐years	12.4	12.6	8.2	13.5	13.7	9.7
Unadjusted hazard ratio (95% CI)		0.99 (0.85–1.15), *p* = 0.891	1.52 (1.30–1.77), *p* < 0.001		0.98 (0.77–1.23), *p* = 0.837	1.39 (1.10–1.76), *p* = 0.006
Adjusted hazard ratio^b^ (95% CI)		0.98 (0.83–1.14), *p* = 0.755	1.40 (1.19–1.64), *p* < 0.001		0.94 (0.74–1.20), *p* = 0.632	1.29 (1.01–1.64), *p* = 0.038

^a^ Incident idiopathic intracranial hypertension and corresponding controls.

^b^ Adjusted for age category, body mass index category, Townsend quintile, smoking status, eating disorder, severe mental illness, back pain, osteoarthritis, rheumatoid arthritis, fibromyalgia, epilepsy, obstructive sleep apnea, polycystic ovary syndrome.

A total of 2984 women with IIH, 27,024 migraine controls and 30,338 population controls were included in the analysis for new onset anxiety. Crude incidence of anxiety was 12.4, 12.6 and 8.2 per 1000 person‐years in the IIH, migraine control and population control groups, respectively (Table 2). Compared to migraine controls, the aHR for anxiety in women with IIH was 0.98 (95% CI 0.83–1.14); compared to population controls, the aHR was 1.40 (95% CI 1.19–1.64). A sensitivity analysis restricted to only women with incident IIH and their corresponding controls made little difference to these results (aHR 0.94, 95% CI 0.74–1.20; and 1.29, 95% CI 1.01–1.64 for IIH compared to migraine and population controls, respectively).

**FIGURE 1:**
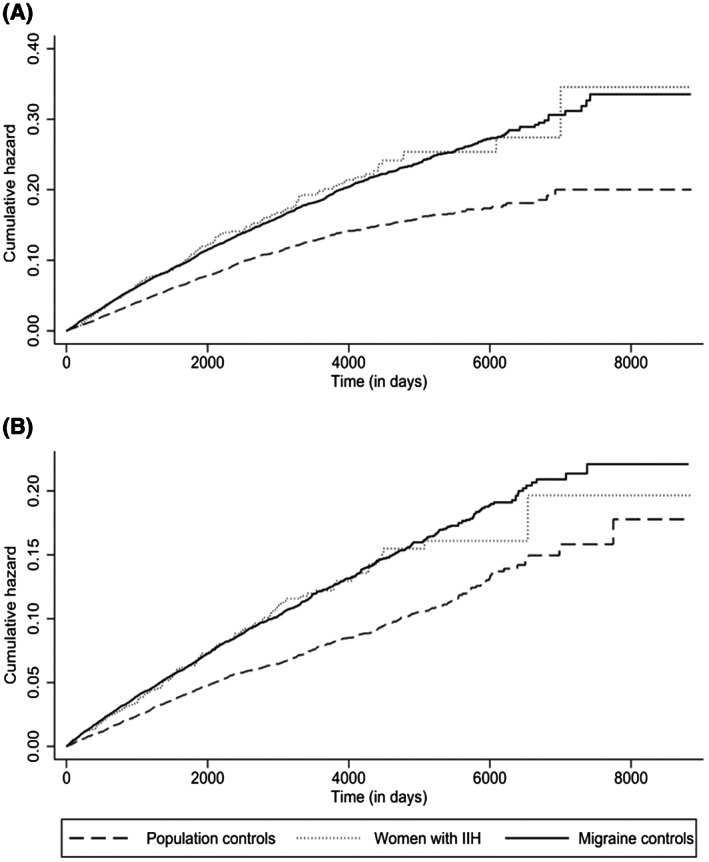
Cumulative hazard of developing (A) depression and (B) anxiety in women with idiopathic intracranial hypertension (IIH), matched control women with migraine, and matched population control women.

Cumulative hazard plots for depression and anxiety outcomes in women with IIH, migraine controls and population controls are shown in Figure 1A,B.

## DISCUSSION

Depression and anxiety are recognized comorbid conditions in those with IIH,^12^, ^13^ and have been shown to predict poorer medical outcomes.^13^, ^17^, ^25^ The mental health burden in IIH has not been previously evaluated in a large population‐based longitudinal cohort study. Depression and anxiety were found to be higher in women with IIH compared to matched population controls, and comparable in women with IIH and matched control women with migraine. The comparable risk of depression and anxiety in women with IIH and matched migraine controls may indicate that presence of headache itself is a possible determinant in these conditions.

Headache is a near universal symptom of IIH, and has recently been shown to be driven by raised ICP.^26^, ^27^, ^28^ When asked to prioritize research in IIH, patients and physicians placed headache as one of the top priorities to understand and manage.^29^ There is currently no licensed treatments for headache in IIH.^6^, ^26^, ^27^ Recently the first prospective open‐label study of a calcitonin gene‐related peptide monoclonal receptor antibody reported substantial improvements in the reduction of monthly moderate/severe headache days.^30^ Topiramate, a migraine preventative therapy, was previously assessed in IIH. This open‐label study evaluated its impact on vision, rather than its beneficial effects on headache disability, and is used off label in routine clinical practice.^6^, ^26^, ^31^ The IIH consensus guidelines recommend topiramate as a useful medication for management of headaches in IIH to avoid medicines such as β‐blockers and tricyclics that may exacerbate weight gain. However, the guidance highlighted caution in those with mental health diagnoses, as it is known to cause depression and increase risk of suicide.^6^

There is an increasing understanding of the pathophysiology of migraine and secondary headache disorders that mimic migraine, like IIH.^32^ There may be a shared biophysiology of migraine and depression with associated low levels of 5‐hydroxytryptamine (5‐HT) or serotonin receptors. There is also genetic evidence of common pathogenetic pathways.^33^ For example, serotonin transporter gene alterations have been identified, with the short allele being associated with risk of depression and increased likelihood of migraine.^34^

Headache is not the only confounding factor in IIH; obesity has been shown to be a major risk factor for development of the condition.^7^, ^8^, ^9^ Both headache and obesity are associated with depression and anxiety. There is a bidirectional relationship between obesity and depression, which has been found to be most pronounced in female adolescents,^35^ an age group where IIH is at its most prevalent.^1^, ^2^ In our study, we found an increased hazard of depression and anxiety in women with IIH compared to population controls that was independent of obesity (controls were matched for BMI). While disability in patients with IIH has previously been found to be predominantly driven by headache,^11^, ^25^, ^36^ headache itself also has a bidirectional relationship with depression.^37^ Therefore assigning independence of depression in IIH is a challenge as it may relate to the underlying disease, the headache, the obesity or all three components.^12^, ^16^ Prescribing headache therapies in IIH with a co‐existing mental health diagnosis is challenging due to polypharmacy and the potential of some medications to cause exacerbation of depression or fatigue.^6^, ^38^ Likewise weight gain is an adverse effect of many antidepressants and indeed antipsychotic medicines, therefore treatment of mental health disorders in IIH needs careful consideration, so as not to exacerbate the disease.^6^

The large sample size in this cohort study is a key strength of this analysis. It has enabled evaluation of the association between mental health conditions (depression and anxiety) and IIH in women, as well as allowing comparison with matched women with migraine. Patients included in IMRD‐UK are generalizable to the UK population. In the UK, diagnosis of IIH is made in the hospital setting; the diagnosis is then communicated to the general practice. However, there is a known risk of diagnostic error in the hospital leading to misclassification bias^39^ and a possibility of data entry error in the hospital or general practice. To further mitigate this, we excluded those with a record of hydrocephalus or cerebral venous sinus thrombosis. The findings are not directly applicable to men with IIH or men with migraine as this study assessed women with IIH, matched women with migraine and population control women. Likewise, the results are not applicable to children aged <16 years with IIH or migraine.

This is the largest national study providing epidemiological data evaluating a depression or anxiety diagnosis for women with IIH and comparing them to matched migraine controls and population controls. The results indicate a high burden of depression and anxiety in IIH similar to that of matched women with migraine. This may suggest the presence of headache may contribute to depression and anxiety in IIH. As those with IIH and a concurrent psychiatric diagnosis have been found to have worse medical outcomes,^16^, ^40^ it is important that clinicians managing women with IIH recognize the increased risk of depression and anxiety in this group so that they can be managed appropriately.

## AUTHOR CONTRIBUTIONS

*Study concept and design*: Krishnarajah Nirantharakumar, Nicola J. Adderley, Alexandra J. Sinclair. *Acquisition of data*: Anuradhaa Subramanian, Krishnarajah Nirantharakumar, Nicola J. Adderley. *Analysis and interpretation of data*: Susan P. Mollan, Anuradhaa Subramanian, Mary Perrins, Nicola J. Adderley, Alexandra J. Sinclair. *Drafting of the manuscript*: Susan P. Mollan, Anuradhaa Subramanian, Nicola J. Adderley. *Revising it for intellectual content*: Susan P. Mollan, Anuradhaa Subramanian, Mary Perrins, Krishnarajah Nirantharakumar, Nicola J. Adderley, Alexandra J. Sinclair. *Final approval of the completed manuscript*: Susan P. Mollan, Anuradhaa Subramanian, Mary Perrins, Krishnarajah Nirantharakumar, Nicola J. Adderley, Alexandra J. Sinclair.

## FUNDING INFORMATION

This study was funded by The Midland Neurosciences and Teaching Research Fund (MNTRF, Registered Charity No. 313446; project reference number: 38). Alexandra J. Sinclair was funded by a National Institute for Health Research (NIHR) clinician scientist fellowship (NIHR‐CS‐011‐028) and the Medical Research Council (MRC), UK (MR/K015184/1) for the duration of the study. Alexandra J. Sinclair is funded by a Sir Jules Thorn Award for Biomedical Science. The views expressed are those of the authors and not necessarily those of the UK National Health Service, NIHR, or the UK Department of Health and Social Care.

Role of Funder/Sponsor: MNTRF, the NIHR and the MRC had no role in the design or conduct of the study; no role in collection, management, analysis, or interpretation of the data; no role in preparation, review, or approval of the manuscript; and no role in the decision to submit the manuscript for publication.

## CONFLICT OF INTEREST

**Susan P. Mollan** reports consulting fees from Invex Therapeutics and Neurodiem; advisory board compensation from Janssen, Santhera, GenSight Biologics and Chugai‐Roche Ltd; and speaker fees from Chiesi, Heidelberg engineering, Allergan, Chugai‐Roche Ltd, Santen, Santhera, Roche and Teva. All conflicts of interest are outside the submitted work. **Anuradhaa Subramanian** reports no competing interests. **Mary Perrins** reports no competing interests. **Krishnarajah Nirantharakumar** reports no competing interests. **Nicola J. Adderley** reports no competing interests. **Alexandra J. Sinclair** reports personal fees from Invex therapeutics in her role as Director with stock holdings, during the conduct of the study; other from Allergan, Novartis, Chiesi and Amgen outside the submitted work.

## ETHICS STATEMENT

Use of IQVIA Medical Research Data (IMRD‐UK) is approved by the UK Research Ethics Committee (reference number: 18/LO/0441); in accordance with this approval, the study protocol was reviewed and approved by an independent Scientific Review Committee (SRC) (reference number: 18THIN070). IMRD‐UK incorporates data from The Health Improvement Network (THIN), A Cegedim Database. Reference made to THIN is intended to be descriptive of the data asset licensed by IQVIA. This work used de‐identified data provided by patients as a part of their routine primary care, therefore written informed consent was not required.

## Supporting information


Appendix S1

## Data Availability

The data used in this study were provided by IQVIA Medical Research Data (IMRD) under license/by permission. Data may be shared on request to the corresponding author with permission of IMRD.
