# Supplementary material for: Depression and anxiety in women with idiopathic intracranial hypertension compared to migraine: A matched controlled cohort study
Source: Headache. 2023 Feb 7;63(2):290–8. doi: 10.1111/head.14465 (PMC10952318; doi:10.1111/head.14465)
Supplement: Supplementary file 1 — Appendix S1 [file HEAD-63-290-s001.docx]

**Supplementary Table 1A. Read code lists for the exposure IIH**

| **Read Code** | **Description** |
| --- | --- |
| F282.00 | Benign intracranial hypertension |
| F282.11 | Pseudotumour cerebri |
| F287.00 | Idiopathic intracranial hypotension |

**Supplementary Table 1B. Read code lists for the exclusion criteria hydrocephalus**

| **Read Code** | **Description** |
| --- | --- |
| 7F1A300 | Drainage of hydrocephalus of fetus to facilitate delivery |
| F113.00 | Acquired communicating hydrocephalus |
| F113000 | Normal pressure hydrocephalus |
| F113011 | Low pressure hydrocephalus |
| F113z00 | Communicating hydrocephalus - acquired NOS |
| F114.00 | Acquired obstructive hydrocephalus |
| F115.00 | Hydrocephalus |
| F117.00 | Infantile posthaemorrhagic hydrocephalus |
| F11X.00 | Post-traumatic hydrocephalus, unspecified |
| F11x300 | Cerebral degeneration due to congenital hydrocephalus |
| FyuA100 | [X]Other hydrocephalus |
| FyuA300 | [X]Hydrocephalus/infectious+parasitic diseases CE |
| FyuA400 | [X]Hydrocephalus in neoplastic disease classified elsewhere |
| FyuA500 | [X]Hydrocephalus in other diseases classified elsewhere |
| FyuAG00 | [X]Post-traumatic hydrocephalus, unspecified |
| L236.00 | Hydrocephalic disproportion |
| L236000 | Hydrocephalic disproportion unspecified |
| L236100 | Hydrocephalic disproportion - delivered |
| L236200 | Hydrocephalic disproportion with antenatal problem |
| L236z00 | Hydrocephalic disproportion NOS |
| L250.12 | Suspect fetal hydrocephaly |
| P10..00 | Spina bifida with hydrocephalus |
| P100.00 | Unspecified spina bifida with hydrocephalus |
| P100000 | Spina bifida with hydrocephalus, unspecified |
| P100100 | Cervical spina bifida with hydrocephalus |
| P100200 | Thoracic spina bifida with hydrocephalus |
| P100300 | Lumbar spina bifida with hydrocephalus |
| P100z00 | Spina bifida with hydrocephalus NOS |
| P102.00 | Spina bifida with hydrocephalus - open |
| P102.11 | Fissured spine with hydrocephalus |
| P102.12 | Hydromyelocele with hydrocephalus |
| P102.13 | Myelocele with hydrocephalus |
| P102.14 | Rachischisis with hydrocephalus |
| P102000 | Unspecified spina bifida with hydrocephalus - open |
| P102100 | Cervical spina bifida with hydrocephalus - open |
| P102200 | Thoracic spina bifida with hydrocephalus - open |
| P102300 | Lumbar spina bifida with hydrocephalus - open |
| P102400 | Sacral spina bifida with hydrocephalus - open |
| P102z00 | Spina bifida with hydrocephalus - open NOS |
| P103.00 | Spina bifida with hydrocephalus - closed |
| P103000 | Unspecified spina bifida with hydrocephalus - closed |
| P103100 | Cervical spina bifida with hydrocephalus - closed |
| P103200 | Thoracic spina bifida with hydrocephalus - closed |
| P103300 | Lumbar spina bifida with hydrocephalus - closed |
| P103400 | Sacral spina bifida with hydrocephalus - closed |
| P103z00 | Spina bifida with hydrocephalus - closed NOS |
| P103z11 | Thoracolumbar spina bifida with hydrocephalus - closed |
| P104.00 | Spina bifida with hydrocephalus of late onset |
| P10y.00 | Other specified spina bifida with hydrocephalus |
| P10yz00 | Other spina bifida with hydrocephalus NOS |
| P10z.00 | Spina bifida with hydrocephalus NOS |
| P23..00 | Congenital hydrocephalus |
| P230.11 | Hydrocephauls with anomaly of aqueduct of Sylvius |
| P233.12 | Hydrocephalus with atresia of foramina of Magendie+Luschka |
| P235.00 | X-linked hydrocephalus |
| P23y.00 | Other specified congenital hydrocephalus |
| P23z.00 | Congenital hydrocephalus NOS |
| Pyu0100 | [X]Other congenital hydrocephalus |
| Pyu0400 | [X]Unspecified spina bifida with hydrocephalus |
| Q402311 | Congenital hydrocephalus due to toxoplasmosis |
| Q48H.00 | Acquired hydrocephalus newborn |

**Supplementary Table 1C. Read code lists for the exclusion criteria cerebral venous thrombosis**

| **Read Code** | **Description** |
| --- | --- |
| G640.00 | Cerebral thrombosis |
| G676.00 | Nonpyogenic venous sinus thrombosis |
| G676000 | Cereb infarct due cerebral venous thrombosis, nonpyogenic |
| G67A.00 | Cerebral vein thrombosis |
| L417.00 | Obstetric cerebral venous thrombosis |
| L417000 | Cerebral venous thrombosis in pregnancy |
| L417100 | Cerebral venous thrombosis in the puerperium |

**Supplementary Table 1D. Read code lists for the migraine controls**

| **Read Code** | **Description** |
| --- | --- |
| 1967 | Abdominal migraine - symptom |
| 8B6N.00 | Migraine prophylaxis |
| F26..00 | Migraine |
| F260.00 | Classical migraine |
| F260.11 | Migraine with aura |
| F261.00 | Common migraine |
| F261.11 | Migraine without aura |
| F261000 | Atypical migraine |
| F261100 | Sick headache |
| F261z00 | Common migraine NOS |
| F262.00 | Migraine variants |
| F262200 | Abdominal migraine |
| F262300 | Basilar migraine |
| F262400 | Ophthalmic migraine |
| F262500 | Periodic migrainous neuralgia |
| F262800 | Migraine induced by oestrogen contraceptive |
| F262z00 | Migraine variant NOS |
| F26y.00 | Other forms of migraine |
| F26y000 | Hemiplegic migraine |
| F26y100 | Ophthalmoplegic migraine |
| F26y200 | Status migrainosus |
| F26y300 | Complicated migraine |
| F26yz00 | Other forms of migraine NOS |
| F26z.00 | Migraine NOS |
| Fyu5300 | [X]Other migraine |
| K584.11 | Migraine - menstrual |
| R090D00 | [D]Abdominal migraine |

**Supplementary Table 1E. Read code lists for depression**

| **Read Code** | **Description** |
| --- | --- |
| E112.00 | Single major depressive episode |
| E112.11 | Agitated depression |
| E112.12 | Endogenous depression first episode |
| E112.13 | Endogenous depression first episode |
| E112.14 | Endogenous depression |
| E112000 | Single major depressive episode, unspecified |
| E112100 | Single major depressive episode, mild |
| E112200 | Single major depressive episode, moderate |
| E112300 | Single major depressive episode, severe, without psychosis |
| E112400 | Single major depressive episode, severe, with psychosis |
| E112500 | Single major depressive episode, partial or unspec remission |
| E112600 | Single major depressive episode, in full remission |
| E112z00 | Single major depressive episode NOS |
| E113.00 | Recurrent major depressive episode |
| E113.11 | Endogenous depression - recurrent |
| E113000 | Recurrent major depressive episodes, unspecified |
| E113100 | Recurrent major depressive episodes, mild |
| E113200 | Recurrent major depressive episodes, moderate |
| E113300 | Recurrent major depressive episodes, severe, no psychosis |
| E113400 | Recurrent major depressive episodes, severe, with psychosis |
| E113500 | Recurrent major depressive episodes,partial/unspec remission |
| E113600 | Recurrent major depressive episodes, in full remission |
| E113700 | Recurrent depression |
| E113z00 | Recurrent major depressive episode NOS |
| E118.00 | Seasonal affective disorder |
| E11y200 | Atypical depressive disorder |
| E11z200 | Masked depression |
| E130.00 | Reactive depressive psychosis |
| E135.00 | Agitated depression |
| E291.00 | Prolonged depressive reaction |
| E2B..00 | Depressive disorder NEC |
| E2B1.00 | Chronic depression |
| Eu32.00 | [X]Depressive episode |
| Eu32.11 | [X]Single episode of depressive reaction |
| Eu32.12 | [X]Single episode of psychogenic depression |
| Eu32.13 | [X]Single episode of reactive depression |
| Eu32000 | [X]Mild depressive episode |
| Eu32100 | [X]Moderate depressive episode |
| Eu32200 | [X]Severe depressive episode without psychotic symptoms |
| Eu32211 | [X]Single episode agitated depressn w'out psychotic symptoms |
| Eu32212 | [X]Single episode major depression w'out psychotic symptoms |
| Eu32213 | [X]Single episode vital depression w'out psychotic symptoms |
| Eu32300 | [X]Severe depressive episode with psychotic symptoms |
| Eu32311 | [X]Single episode of major depression and psychotic symptoms |
| Eu32312 | [X]Single episode of psychogenic depressive psychosis |
| Eu32313 | [X]Single episode of psychotic depression |
| Eu32314 | [X]Single episode of reactive depressive psychosis |
| Eu32400 | [X]Mild depression |
| Eu32500 | [X]Major depression, mild |
| Eu32600 | [X]Major depression, moderately severe |
| Eu32700 | [X]Major depression, severe without psychotic symptoms |
| Eu32800 | [X]Major depression, severe with psychotic symptoms |
| Eu32y00 | [X]Other depressive episodes |
| Eu32y11 | [X]Atypical depression |
| Eu32y12 | [X]Single episode of masked depression NOS |
| Eu32z00 | [X]Depressive episode, unspecified |
| Eu32z11 | [X]Depression NOS |
| Eu32z12 | [X]Depressive disorder NOS |
| Eu32z13 | [X]Prolonged single episode of reactive depression |
| Eu32z14 | [X] Reactive depression NOS |
| Eu33.00 | [X]Recurrent depressive disorder |
| Eu33.11 | [X]Recurrent episodes of depressive reaction |
| Eu33.12 | [X]Recurrent episodes of psychogenic depression |
| Eu33.13 | [X]Recurrent episodes of reactive depression |
| Eu33.14 | [X]Seasonal depressive disorder |
| Eu33.15 | [X]SAD - Seasonal affective disorder |
| Eu33000 | [X]Recurrent depressive disorder, current episode mild |
| Eu33100 | [X]Recurrent depressive disorder, current episode moderate |
| Eu33200 | [X]Recurr depress disorder cur epi severe without psyc sympt |
| Eu33211 | [X]Endogenous depression without psychotic symptoms |
| Eu33212 | [X]Major depression, recurrent without psychotic symptoms |
| Eu33214 | [X]Vital depression, recurrent without psychotic symptoms |
| Eu33300 | [X]Recurrent depress disorder cur epi severe with psyc symp |
| Eu33311 | [X]Endogenous depression with psychotic symptoms |
| Eu33313 | [X]Recurr severe episodes/major depression+psychotic symptom |
| Eu33314 | [X]Recurr severe episodes/psychogenic depressive psychosis |
| Eu33315 | [X]Recurrent severe episodes of psychotic depression |
| Eu33316 | [X]Recurrent severe episodes/reactive depressive psychosis |
| Eu33400 | [X]Recurrent depressive disorder, currently in remission |
| Eu33y00 | [X]Other recurrent depressive disorders |
| Eu33z00 | [X]Recurrent depressive disorder, unspecified |
| Eu33z11 | [X]Monopolar depression NOS |
| Eu34100 | [X]Dysthymia |

**Supplementary Table 1F. Read code lists for anxiety**

| **Read Code** | **Description** |
| --- | --- |
| 146G.00 | H/O: agoraphobia |
| 8CAZ000 | Patient given advice about management of anxiety |
| 8HHp.00 | Referral for guided self-help for anxiety |
| E20..00 | Neurotic disorders |
| E200.00 | Anxiety states |
| E200000 | Anxiety state unspecified |
| E200100 | Panic disorder |
| E200200 | Generalised anxiety disorder |
| E200400 | Chronic anxiety |
| E200500 | Recurrent anxiety |
| E200z00 | Anxiety state NOS |
| E202.00 | Phobic disorders |
| E202.11 | Social phobic disorders |
| E202.12 | Phobic anxiety |
| E202000 | Phobia unspecified |
| E202100 | Agoraphobia with panic attacks |
| E202200 | Agoraphobia without mention of panic attacks |
| E202300 | Social phobia, fear of eating in public |
| E202400 | Social phobia, fear of public speaking |
| E202500 | Social phobia, fear of public washing |
| E202600 | Acrophobia |
| E202700 | Animal phobia |
| E202800 | Claustrophobia |
| E202900 | Fear of crowds |
| E202B00 | Cancer phobia |
| E202C00 | Dental phobia |
| E202E00 | Fear of pregnancy |
| E202z00 | Phobic disorder NOS |
| E20y.00 | Other neurotic disorders |
| E20y200 | Other occupational neurosis |
| E20y300 | Psychasthenic neurosis |
| E20yz00 | Other neurotic disorder NOS |
| E20z.00 | Neurotic disorder NOS |
| E28..00 | Acute reaction to stress |
| E280.00 | Acute panic state due to acute stress reaction |
| E281.00 | Acute fugue state due to acute stress reaction |
| E282.00 | Acute stupor state due to acute stress reaction |
| E283.00 | Other acute stress reactions |
| E283100 | Acute posttrauma stress state |
| E283z00 | Other acute stress reaction NOS |
| E284.00 | Stress reaction causing mixed disturbance of emotion/conduct |
| E28z.00 | Acute stress reaction NOS |
| E28z.12 | Flying phobia |
| Eu22y11 | [X]Delusional dysmorphophobia |
| Eu4..00 | [X]Neurotic, stress - related and somoform disorders |
| Eu40.00 | [X]Phobic anxiety disorders |
| Eu40000 | [X]Agoraphobia |
| Eu40011 | [X]Agoraphobia without history of panic disorder |
| Eu40012 | [X]Panic disorder with agoraphobia |
| Eu40100 | [X]Social phobias |
| Eu40111 | [X]Anthropophobia |
| Eu40112 | [X]Social neurosis |
| Eu40200 | [X]Specific (isolated) phobias |
| Eu40211 | [X]Acrophobia |
| Eu40212 | [X]Animal phobias |
| Eu40213 | [X]Claustrophobia |
| Eu40214 | [X]Simple phobia |
| Eu40300 | [X]Needle phobia |
| Eu40y00 | [X]Other phobic anxiety disorders |
| Eu40z00 | [X]Phobic anxiety disorder, unspecified |
| Eu40z11 | [X]Phobia NOS |
| Eu40z12 | [X]Phobic state NOS |
| Eu41.00 | [X]Other anxiety disorders |
| Eu41000 | [X]Panic disorder [episodic paroxysmal anxiety] |
| Eu41100 | [X]Generalized anxiety disorder |
| Eu41111 | [X]Anxiety neurosis |
| Eu41112 | [X]Anxiety reaction |
| Eu41113 | [X]Anxiety state |
| Eu41300 | [X]Other mixed anxiety disorders |
| Eu41y00 | [X]Other specified anxiety disorders |
| Eu41y11 | [X]Anxiety hysteria |
| Eu41z00 | [X]Anxiety disorder, unspecified |
| Eu41z11 | [X]Anxiety NOS |
| Eu42.11 | [X]Anankastic neurosis |
| Eu42.12 | [X]Obsessive-compulsive neurosis |
| Eu43.00 | [X]Reaction to severe stress, and adjustment disorders |
| Eu43000 | [X]Acute stress reaction |
| Eu43012 | [X]Acute reaction to stress |
| Eu43y00 | [X]Other reactions to severe stress |
| Eu43z00 | [X]Reaction to severe stress, unspecified |
| Eu45212 | [X]Dysmorphophobia nondelusional |
| Eu45215 | [X]Nosophobia |
| Eu51511 | [X]Dream anxiety disorder |
| Z481.00 | Phobia counselling |
| Z4L1.00 | Anxiety counselling |
| Z522400 | Desensitisation - phobia |
| Z522600 | Flooding - obsessional compulsive disorder |
| Z522700 | Flooding - agoraphobia |

**Supplementary Table 2. Baseline characteristics of exposed participants with idiopathic intracranial hypertension (IIH) and propensity score-matched controls with migraine (but without IIH), with standardised mean differences (SMD)**

|  | **Primary Analysis** | | | **Sensitivity Analysis** | | |
| --- | --- | --- | --- | --- | --- | --- |
|  | **Exposed** | **Migraine controls** | **SMD** | **Exposed** | **Migraine controls** | **SMD** |
|  | **(n=3,411)** | **(n=30,879)** |  | **(n=1,555)** | **(n=13,966)** |  |
| IIH Duration (prevalent patients), years [Mean (SD)] | 8.5 (8.9) | N/A |  | N/A | N/A |  |
| Age at IIH diagnosis, years [Mean (SD)] | 29.4 (11.5) | N/A |  | 32.1 (11.4) | N/A |  |
| Age, years [Mean (SD)] | 34.0 (12.7) | 33.7 (11.8) | 0.031 | 32.1 (11.4) | 31.9 (10.7) | 0.026 |
| BMI, kg/m^2^ [Mean (SD)] | 34.9 (8.1) | 28.8 (6.3) | 0.845 | 35.7 (8.1) | 29.4 (6.4) | 0.856 |
| Smoking status, n (%) |  |  | 0.105 |  |  | 0.158 |
| Non-smoker | 1657 (48.6) | 16308 (52.8) |  | 789 (50.7) | 7353 (52.6) |  |
| Ex-smoker | 584 (17.1) | 4791 (15.5) |  | 267 (17.2) | 2197 (15.7) |  |
| Smoker | 976 (28.6) | 7747 (25.1) |  | 457 (29.4) | 3650 (26.1) |  |
| Missing | 194 (5.7) | 2033 (6.6) |  | 42 (2.7) | 766 (5.5) |  |
| Townsend deprivation quintile, n (%) |  |  | 0.120 |  |  | 0.109 |
| 1 (Least deprived) | 439 (12.9) | 4805 (15.6) |  | 201 (12.9) | 1991 (14.3) |  |
| 2 | 434 (12.7) | 4543 (14.7) |  | 188 (12.1) | 2023 (14.5) |  |
| 3 | 601 (17.6) | 5616 (18.2) |  | 277 (17.8) | 2529 (18.1) |  |
| 4 | 619 (18.1) | 5423 (17.6) |  | 282 (18.1) | 2518 (18.0) |  |
| 5 (Most deprived) | 515 (15.1) | 4103 (13.3) |  | 262 (16.8) | 1939 (13.9) |  |
| Missing | 803 (23.5) | 6389 (20.7) |  | 345 (22.2) | 2966 (21.2) |  |
| Ethnicity, n (%) |  |  | 0.064 |  |  | 0.080 |
| White | 1766 (51.8) | 15389 (49.8) |  | 754 (48.5) | 7181 (51.4) |  |
| South Asian | 31 (0.9) | 447 (1.4) |  | 19 (1.2) | 195 (1.4) |  |
| Black Afro-Caribbean | 60 (1.8) | 484 (1.6) |  | 33 (2.1) | 242 (1.7) |  |
| Mixed Race | 15 (0.4) | 162 (0.5) |  | 6 (0.4) | 73 (0.5) |  |
| Chinese/middle eastern/other | 15 (0.4) | 156 (0.5) |  | 5 (0.3) | 76 (0.5) |  |
| Missing | 1524 (44.7) | 14241 (46.1) |  | 738 (47.5) | 6199 (44.4) |  |
| Comorbidities, n (%) |  |  |  |  |  |  |
| Back pain | 780 (22.9) | 6522 (21.1) | 0.042 | 418 (26.9) | 2747 (19.7) | 0.171 |
| Polycystic ovary syndrome | 253 (7.4) | 1223 (4.0) | 0.150 | 123 (7.9) | 621 (4.4) | 0.144 |
| Osteoarthritis | 138 (4.0) | 914 (3.0) | 0.059 | 42 (2.7) | 287 (2.1) | 0.042 |
| Epilepsy | 90 (2.6) | 603 (2.0) | 0.046 | 32 (2.1) | 294 (2.1) | 0.003 |
| Fibromyalgia | 69 (2.0) | 354 (1.1) | 0.070 | 36 (2.3) | 170 (1.2) | 0.083 |
| Eating Disorder | 63 (1.8) | 514 (1.7) | 0.014 | 26 (1.7) | 241 (1.7) | 0.004 |
| Severe Mental Illness | 55 (1.6) | 238 (0.8) | 0.078 | 27 (1.7) | 115 (0.8) | 0.081 |
| Obstructive sleep apnoea | 34 (1.0) | 95 (0.3) | 0.086 | 24 (1.5) | 39 (0.3) | 0.133 |
| Rheumatoid Arthritis | 21 (0.6) | 154 (0.5) | 0.016 | 10 (0.6) | 48 (0.3) | 0.043 |

SMD = standardized mean difference, SD = standard deviation, BMI = body mass index

15,837,846 patients eligible from the selected practices

Eligible patients aged 16 and above (n= 9,345,096)

Patients with IIH

(n=3,931)

Exposed

(n=3,411)

Patients with migraine

(n=610,577)

Propensity Score Matching

Per exposed patient, up to 10

age, sex, BMI and health authority region matched population controls

(n=38,660)

Exclusion criteria:

1. Diagnosis of IIH (n=1,079)
2. Men (n=167,074)
3. Diagnosis of CVT (n=76)
4. Diagnosis of hydrocephalus (n=295)

Exclusion criteria:

1. Men (n=467)
2. Diagnosis of CVT (n=23)
3. Diagnosis of hydrocephalus (n=30)

Exclusion criteria:

1. Men (n=4,609)
2. Diagnosis of CVT (n=2)
3. Diagnosis of hydrocephalus (n=25)
4. Matched to an ineligible IIH patient (n=529)

808 practices eligible for inclusion between 1995 to 2019

Population controls

(n=33,495)

Patients with migraine

(n=442,053)

Migraine controls

(n=30,879)

**Supplementary Figure 1. Flow chart showing participant selection.**
